# A Virtual Simulator to Improve Weight-Related Communication Skills for Health Care Professionals: Mixed Methods Pre-Post Pilot Feasibility Study

**DOI:** 10.2196/65949

**Published:** 2025-08-15

**Authors:** Fiona Quigley, Leona Ryan, Raymond Bond, Toni McAloon, Huiru Zheng, Anne Moorhead

**Affiliations:** 1 Institute of Nursing and Health Research, School of Communication and Media, Ulster University, 2-24 York Street, Belfast, BT15 1ED, United Kingdom, 44 02871675219; 2 School of Psychology, Ollscoil na Gaillimhe – University of Galway, Galway, Ireland; 3 School of Computing, Ulster University, Belfast, United Kingdom; 4 Institute of Nursing and Health Research, Ulster University, Belfast, United Kingdom

**Keywords:** overweight, weight stigma, weight-related communication, virtual simulation, eLearning, medical education, healthcare communication, doctors, nurses, dietitians, simulator, professionals, healthcare, obesity, HCP, 3D, virtual, VITAL-COMS, tool, nutrition, mixed-method, digital training

## Abstract

**Background:**

Discussing weight remains a sensitive and often avoided topic in health care, despite rising prevalence of obesity and calls for earlier, more compassionate interventions. Many health care professionals report inadequate training and low confidence to discuss weight, while patients often describe feeling stigmatized or dismissed. Digital simulation offers a promising route to build communication skills through supporting repeatable and reflective practice in a safe space. VITAL-COMS (Virtual Training and Assessment for Communication Skills) is a novel simulation tool designed to support health care professionals in navigating weight-related conversations with greater understanding and skill.

**Objective:**

This study aimed to assess the potential of VITAL-COMS as a digital simulation training tool to improve weight-related communication skills among health care professionals.

**Methods:**

A mixed-method feasibility study was conducted online via Zoom (Zoom Video Communications) between January to July 2021, with UK-based nurses, doctors, and dietitians. The intervention comprised educational videos and 2 simulated patient scenarios with real-time verbal interaction. Pre- and posttraining self-assessments of communication skills and conversation length were collected. Participants also completed a feasibility questionnaire. Descriptive statistics were used to analyze the feasibility questionnaire, and open-ended feedback was analyzed using content analysis. Paired-samples *t* tests were used to assess changes in communication skills and conversation length before and post training.

**Results:**

In total, 31 participants completed the study. There was a statistically significant improvement in self-assessed communication skills following training (mean difference=3.9; 95% CI, 2.54‐5.26; *t*_30_=−5.76, *P*=.001, Cohen *d*=1.03). Mean conversation length increased significantly in both scenarios: in the female patient scenario, from 3.73 (SD 1.36) to 6.08 (SD 2.26) minutes, with a mean difference of 2.35 minutes (95% CI, 1.71‐2.99; *t*_30_=7.49, *P*=.001, Cohen *d*=1.34); and in the male scenario, from 3.61 (SD 1.12) to 5.65 (SD 1.76) minutes, a mean difference of 2.03 minutes (95% CI, 1.51‐2.55; *t*_30_=8.03, *P*=.001, Cohen *d*=1.44). Participants rated the simulation positively, with 97% (95% CI 90%‐100%) supporting wider use in health care and 84% (95% CI 71%‐97%) reporting emotional engagement. Content analysis of feedback generated two themes: (1) adapting to this form of learning and (2) recognizing the potential of simulation to support reflective, skills-based training. A minority, 13% (95% CI 1%‐25%) expressed a preference for alternative learning methods.

**Conclusions:**

VITAL-COMS was feasible to implement and acceptable to a diverse group of health care professionals. Participants demonstrated significant improvements in self-assessed communication skills and patient-scenario engagement. The simulation was perceived as realistic, emotionally engaging, and well-suited for training in sensitive conversations. These findings support further development and integration of VITAL-COMS into health education programs. Next steps include the translation of the insights identified in this study to inform a tool supported by generative artificial intelligence.

## Introduction

Obesity is a leading contributor to chronic disease and reduced quality of life [1,2]; yet, weight remains a sensitive and often avoided topic in clinical settings [3,4]. Despite national and global policy goals and clinical guidelines calling for earlier, more compassionate intervention [5-7], many health care professionals (HCPs) report inadequate training, lack of confidence, and fear of damaging the patient relationship when discussing weight [8-11]. Patients, in turn, often describe feeling stigmatized, misunderstood, or dismissed when seeking support for weight-related concerns [12-15] Addressing this training gap requires more than knowledge transfer—it requires practical, reflective skills development in how to talk about weight [16-18].

Digital or virtual simulation offers a flexible and scalable route to delivering communication skills training [19-21]. Simulation-based training enables learners to rehearse conversations in a safe environment and build confidence through repetition, feedback, and debrief [22]. When designed with behavioral science and communication theory, virtual simulations can foster the deeper understanding needed to engage patients with empathy and nuance [23-25]. For weight-related communication, where misalignment between HCP intention and patient perception is common [26-28], virtual simulation tools offer a safe space to surface and reflect on these tensions.

VITAL-COMS (Virtual Training and Assessment for Communication Skills) was developed to address the misalignments between HCPs and patients when discussing weight. The tool presents HCPs with simulated patient scenarios to practice sensitive, weight-related conversations, guided by principles from the COM-B (communication, opportunity, and motivation) model to support behavior change [23], Hargie’s skilled interpersonal communication model [24], and the theory of deliberate practice [25]. The full development of VITAL-COMS is described elsewhere [26-32]. This study focuses on the preliminary findings of the feasibility study.

VITAL-COMS uses the Wizard of Oz (WoZ) method to control the simulated patient’s responses. In this design technique, a human operator (FQ) manages the system’s output to mimic intelligent responses that do not yet exist technologically [33,34]. WoZ allows designers to prototype complex interactions and gather authentic user feedback without the cost or limitations of fully developed artificial intelligence (AI) [35]. WoZ allows design ideas to be tested when the technology and AI do not yet exist or if it would take too much time or expense to explore a new idea. The virtual patient character in the simulator is operated by the primary author (FQ) using a WoZ controller designed for the simulation. In VITAL-COMS, this means tailoring verbal feedback from the virtual patient in real time to match the learner’s communication, offering a high-fidelity, emotionally resonant training experience. More detail on the WoZ controller and how VITAL-COMS works can be found in MultimediaAppendices 1  2.

WoZ methods have been successfully used in health care education to simulate patient interactions, particularly where natural language, emotion, and timing are crucial [36]. This approach offers a flexible and iterative way to explore what kinds of digital communication training are most effective, while also revealing learners’ needs and reactions [37]. As HCPs continue to navigate the challenges of supporting patients living with obesity, tools like VITAL-COMS provide a timely and evidence-based approach to addressing the training gaps.

### Aim

The aim of this pilot feasibility study was to assess the potential of the VITAL-COMS digital simulation training tool to improve weight-related communication skills among HCPs.

### Research Questions

This feasibility study addresses the following research questions Textbox 1:

Textbox 1. **Primary and secondary questions.**

Primary research question

- Does the VITAL-COMS (virtual training and assessment for communication skills) virtual simulator improve weight-related health communication skills?

Secondary research questions

- What is the perceived fidelity of the Wizard of Oz–based simulation from the participants’ perspective?
- What is the perspective on VITAL-COMS leading to improvement in health care practice?

## Methods

### Design

A pre-post, mixed methods feasibility study was conducted to evaluate the viability of a virtual simulator designed to enhance weight-related health communication skills (VITAL-COMS). The study is reported following the CONSORT (Consolidated Standards of Reporting Trials) statement: extension to pilot and feasibility trials [38].

### Technical Design

The VITAL-COMS virtual simulator was developed using the Unity 3D game engine (version 2019.3.5f). Development incorporated a suite of Unity’s C# libraries to construct the user interface and implement speech simulation for the virtual patients. 3D character models were generated using Adobe Mixamo, and the Unity add-on components SALSA Lip-Sync, EmoteR, and Eyes were integrated to facilitate realistic speech articulation and emotional expression in the virtual patients. The tool comprised a pretraining stage of watching short educational videos on obesity science, weight-related communication, and weight stigma, then using the simulation tool (MultimediaAppendices 1  2).

### Participants and Recruitment

This study used purposive and snowball sampling strategies to obtain a sample of 31 participants. This sample size aligns with recommendations for feasibility trials, which suggest total sample sizes ranging from N=24 to 50 [39]. The overall sample comprised both UK-based undergraduate and postgraduate allied health professionals and qualified doctors, nurses, and dietitians. Undergraduate and postgraduate students (n=2) were recruited via an email distributed by course directors at Ulster University. Targeted courses included undergraduate Nursing, Physiotherapy, Dietetics, and Occupational Therapy, as well as the MSc in Advanced Practice Nursing. Eligibility for participation was determined by enrollment in courses with curricula specifically addressing lifestyle factors related to the prevention, management, and recovery of ill health.

Qualified doctors, nurses, and dietitians (n=29) were recruited using purposive and snowball sampling. Eligibility for professional participants was contingent upon current practice within United Kingdom primary or secondary care settings and documented clinical experience in weight management counseling. Recruitment involved a social media advertisement posted on Ulster University’s Twitter (currently known as X) and Facebook (Meta) accounts. The advertisement was subsequently disseminated through snowball sampling by tagging UK-based health care organizations and obesity charities. All participants provided informed consent before participation.

### Procedure and Materials

#### Quantitative Data Collection

Upon obtaining informed consent, a participation date was arranged. Participants were subsequently provided with a secure, password-protected Zoom (Zoom Video Communications) meeting link via email before the scheduled session. Participants were invited to complete a brief demographic questionnaire, “About You.” Participants also completed a self-reported knowledge assessment questionnaire on weight-related communication skills. The questionnaire was developed in relation to the existing literature and piloted with an advisory group (Multimedia Appendix 3).

The training intervention was delivered in 2 sequential parts, with participants offered the option of a brief break between sessions. In part 1, participants engaged in simulated consultations with 2 virtual patient scenarios, 1 male and 1 female. During these scenarios, participants interacted verbally with the virtual patient characters. In part 2, participants repeated the same scenarios, but with the addition of supplementary educational videos designed to enhance communication skills within each scenario (MultimediaAppendices 4  5). Following the completion of part 2, participants repeated the knowledge assessment questionnaire to evaluate potential changes in self-reported weight-related communication knowledge and skill levels. The temporal data associated with the primary author’s activation of the WoZ controller buttons were recorded to facilitate an analysis of the author’s controller operation during the simulated patient interactions.

The training concluded with a feasibility questionnaire, adapted from the technology acceptance model [40] and the digital simulation literature. Piloted and refined with the advisory group, it included yes or no items such as “I’d like to see this type of training used more in healthcare” and “I can see other uses for this type of training.” Feasibility was assessed by calculating the percentage of “yes” responses.

#### Qualitative Data Collection

The feasibility questionnaire included one open-ended question, where participants were asked to add any other thoughts on the training.

### Data Management and Analysis

#### Quantitative Data

Descriptive statistics were calculated for participant characteristics at baseline, including demographic data from the “About You” questionnaire, feasibility questionnaire responses, and WoZ controller button activation timestamps. All questionnaire data were collected online via the Qualtrics survey platform. To address the aims of the feasibility study, the primary focus was on descriptive data. However, exploratory significance testing using paired-samples *t* tests was conducted to evaluate within-group mean differences in weight-related communication skills between pre- and posttraining knowledge self-assessment responses. Data were analyzed using SPSS (version 28; IBM Corp, 2021).

#### Qualitative Data

Qualitative data from the open-ended feasibility questionnaire responses were analyzed using a systematic content analysis methodology [41]. The primary author initiated the analysis by familiarization with the textual data. A coding system was developed, informed by the initial patterns identified within the data. This coding scheme was then applied to code the data, facilitating the identification of recurrent themes and patterns. The identified themes were reviewed with another author (LR) to ensure alignment with the raw data. Finally, these themes were interpreted to explain key participant perspectives and experiences.

### Ethical Considerations

Ethical approval for this study was granted by the Ulster University Communications and Media Ethics Filter Committee in December 2020 (reference number: CMFC-20-012). Signed, written informed consent was obtained from all participants via email after they responded to study advertisements and confirmed eligibility. Participants completed the study in private settings of their choosing via Zoom. Data from the online Zoom meetings were transcribed from downloaded audio recordings and anonymized using participant identification numbers. All identifiable information was removed, and data were stored securely in password-protected files accessible only to the research team. No compensation was provided to participants.

## Results

### Overview

Participant attrition was observed during the feasibility study. Among student participants, a 33% dropout rate occurred between the consent and completion stages. For HCPs, a 68% attrition rate was noted from initial expression of interest via social media to the provision of informed consent. Among those HCPs who provided consent, 20% did not complete the study. Participant reports indicated that the COVID-19 pandemic significantly impacted their available time, contributing to attrition. In addition, some participants reported a perceived lack of content knowledge influencing their decision not to complete the study.

### Participant Characteristics

A total of 31 participants, comprising a UK-wide cohort of male and female nurses, doctors, dietitians with varying levels of professional experience, and dietetic students from Ulster University, completed the feasibility study (Table 1). The sample was predominantly female (29/31, 94%) and exhibited a significant proportion of participants with over 10 years of professional practice (21/31, 68%).

**Table 1.** Characteristics of participants who completed the feasibility study.

Participant characteristics and professions		Participants, n (%)
Profession		
	Nurse	8 (26)
	Doctor	9 (29)
	Dietitian	12 (39)
	Student (dietitian)	2 (6)
Gender		
	Male	2 (6)
	Female	29 (94)
Years in practice		
	1‐5	6 (20)
	6‐10	2 (6)
	10+	21 (68)
	Student (dietitian)	2 (6)
Work area		
	Primary care	16 (52)
	Secondary care	9 (29)
	Other	4 (13)
Location		
	Northern Ireland	9 (29)
	Scotland	4 (13)
	Wales	2 (6)
	England	16 (52)
Previous training or experiences in weight management		
	Yes	19 (61)
	No	12 (39)
Previous experiences of eLearning simulations		
	Yes	8 (26)
	No	23 (74)

### Descriptive Statistics

All dietitians (12/12, 100%) reported previous training in weight management. Few doctors (2/9, 23%) and 50% (4/8) of nurses reported training in weight management. Dietitians and nurses were more likely to have reported training in weight management than doctors. Students were less likely to participate in the study (2/31, 6%). Only 26% (8/31) of participants had previous experience with simulation-based learning. Most participants were from England (16/31, 52%), while the smallest proportion came from Wales (2/31, 6%).

Participants' usage time for the VITAL-COMS tool ranged from 18.1 to 41.4 minutes. The mean duration was 24.6 (SD 5.4, median 24.0) minutes. Most participants completed the tool within 20 to 30 minutes, though one participant took over 40 minutes, which may represent a mild outlier.

### Inferential Statistics

A paired-samples *t* test revealed a statistically significant improvement in participants’ self-assessed weight-related communication skills following training (*t*_30_=−5.76, *P*=.001). Specifically, mean scores increased from 28.36 (SD 7.6) pretraining to 32.35 (SD 5.7) post-training, with a mean difference of 3.9 (SD 3.7; 95% CI 2.54‐5.6). The effect size, as measured by Cohen *d*, was 1.03, indicating a strong effect. Doctors and nurses demonstrated the most substantial improvements, while dietitians showed the least.

Paired-samples *t* tests revealed statistically significant increases in conversation length following training for both the female (*P*=.001) and male virtual patient scenarios (*P*=.001). For the female scenario, the mean conversation length increased from 3.73 (SD 1.36) minutes pretraining to 6.08 (SD 2.26) minutes post training, representing a mean difference of 2.35 (SD 1.75, 95% CI 1.73‐2.97) minutes. This increase was statistically significant (*t*_30_=7.49, *P*=.001) with a large effect size (Cohen *d*=1.34). Similarly, for the male scenario, the mean conversation length increased from 3.61 (SD 1.12) minutes pretraining to 5.65 (SD 1.76) minutes post training, with a mean difference of 2.03 (SD 1.41, 95% CI 1.53‐2.53) minutes. This increase was also statistically significant (*t*_30_=8.03, *P*=.001) and demonstrated a large effect size (Cohen *d*=1.44).

### Fidelity of VITAL-COMS and the WoZ-Based Simulator

Following completion of the feasibility questionnaire, participants were invited to provide open-ended feedback on their experience with VITAL-COMS. Content analysis of the 84% (26/31) of participants who responded yielded two overarching themes: (1) “Getting used to this type of learning” and (2) “What this type of learning can do for people.”

#### “Getting Used to This Type of Learning”

Participants generally reported positive reactions to the VITAL-COMS tool, though many noted an initial adjustment period. Some participants could immediately engage, while others found the lack of facial expression in the characters difficult to adjust to. Participants perceived a favorable balance between realism and learning stimulation, as illustrated by the comment

*An enjoyable experience – enough verisimilitude to be meaningful and to stimulate learning, but not so real that it felt uncomfortable or threatening.*

[Doctor, male]

This balance is referred to as effortful learning and is often a criticism of poorly designed eLearning content – that is, poorly designed digital learning does not challenge the user enough. Participants also reported initial feelings of awkwardness, stating, “It was very different to anything I have done before and I felt awkward, but if more used to it, I think it would be really useful” (Nurse, female). Despite this, the tool’s overall effectiveness was consistently acknowledged.

#### “What This Training Does for People”

Participants reflected on who might benefit most from the tool, how the tool might help them to think and reflect, and the difference between this type of training and classroom-based training. Training for undergraduate students was mentioned frequently as a potential use for the tool. This seemed to be because it helped participants to practice sensitive conversations without the risk of failure in real life -

*I think it is great to have this type of training as a “no risk” forum for practicing sensitive topics - the opportunity to do so has been limited to ’real life’ where there is often more harm than good whilst people are upskilling.*

[Nurse, female]

Mentions of reflection and making participants think were common from most participants. From the educational videos, the use of examples and good questions to ask was valued as a practical approach to engage with patients. The scenario-based learning approach and being able to try again seemed particularly helpful to prompt thinking and reflection,

*Interesting to do this. Made me think about what I was saying and also already thinking about how I would respond differently again.*

[Dietitian, female]

Comparisons between this type of virtual training and classroom training were drawn by many participants. Some felt that the addition of a facilitator for group discussions would add to the realism and ability to reflect. Participants suggested a combined or blended learning approach might be useful:

*I think it would be good to have in combination with other training as we miss the emotional/ feelings and body language side which is important.*

[Dietitian, female]

### Participants’ Perspective of the Feasibility Study

Poststudy feasibility questionnaire results indicated a predominantly positive reception among participants. Specifically, a substantial majority expressed a desire for increased usage of such tools within health care settings (97%, 95% CI 91%‐100%) and identified potential applications beyond the study context (97%, 95% CI 91%‐100%) Furthermore, 90% (95% CI 80%‐100%) of participants anticipated colleagues benefiting from the tool’s implementation. Notably, 84% (95% CI 71%‐97%) reported experiencing emotional engagement during the simulation, suggesting a high degree of perceived fidelity in the learning experience. The use of simulated training for sensitive topics, such as weight management, was favorably received by 84% (95% CI 71%‐97%) of participants. While a majority (74%, 95% CI 59%‐90%) preferred scenario-based simulated learning for skills development, a minority, 13% (95% CI, 1%‐25%) expressed a general dislike for this pedagogical approach. In addition, 19% (95% CI 5%‐33%) indicated a preference for alternative learning modalities.

## Discussion

### Principal Findings

This study evaluated the feasibility and preliminary efficacy of VITAL-COMS, a previously developed and usability-tested weight-related communication training tool for HCPs. Participants demonstrated statistically significant improvements in self-assessed weight-related communication skills following training with VITAL-COMS. This was accompanied by a significant increase in the duration and engagement level of conversations with virtual characters. Specifically, doctors and nurses exhibited the greatest improvement, likely due to their comparatively limited previous formal training in this domain, while dietitians, who possess greater baseline expertise in weight-related communication, showed smaller, albeit still statistically significant, gains. These findings suggest VITAL-COMS is effective in enhancing both communication confidence and consultative engagement among health care professionals.

Most participants rated the training as realistic and appropriately challenging. Only 13% of participants reported disliking the training modality, and 19% expressed a preference for alternative learning approaches. Several participants suggested a blended model, combining simulator-based training with subsequent peer or patient role-play. This highlights the importance of adaptable delivery formats to optimize engagement and satisfaction. The tool’s real-time feedback functionality contributed to longer, more detailed conversations, demonstrating its potential for direct application in real-world clinical settings.

The observed lower levels of improvement among dietitians may reflect their pre-existing confidence and familiarity with weight-related communication. Consequently, future iterations of VITAL-COMS should incorporate more advanced content tailored to meet specific developmental needs of this professional group.

Low student participation, likely attributable to pandemic-related pressures, academic workloads, and hesitancy to engage with sensitive topics, was a notable limitation. This was corroborated by feedback from participating students. Future iterations could address this by integrating the training into course curricula, offering greater flexibility in scheduling, and providing novice learners with more comprehensive introductory guidance on navigating difficult conversations.

VITAL-COMS directly addresses common barriers to effective weight-related health care communication, particularly misalignment between HCPs’ assumptions and patient needs. Through educational videos and real-time feedback, the tool facilitates the reduction of misunderstandings that can compromise communication quality. Posttraining feasibility questions and feedback interviews indicated that participants engaged in reflexivity, recalling previous patient interactions and identifying emotionally challenging encounters. This process is a recognized driver of behavioral change and quality improvement in health care [42,43].

Furthermore, the tool also addresses weight stigma by drawing participants' attention to the impact of weight stigma in the educational videos (Multimedia Appendix 5). Weight stigma is a well-documented barrier in clinical settings [4,7,16]. By foregrounding patient experiences and modeling empathetic communication, VITAL-COMS aims to mitigate patient anticipation of judgment or internalized weight stigma, both of which contribute to disengagement in weight-related care [44,45].

### Implications of Findings

The VITAL-COMS training offers a precision-based approach and nuanced approach to weight-related communication training, moving beyond traditional weight management messaging such as “eat less, move more.” By fostering the exploration of complex etiological factors contributing to weight gain, including biological, environmental, and psychosocial determinants, VITAL-COMS facilitates a more individualized and respectful patient-centered dialogue. This approach aligns with national and international health policy initiatives, such as the NHS (United Kingdom) health service long-term plan [46] and other international guidelines and calls to action [47-49], which emphasize the importance of enhanced nutrition and weight stigma education among health care professionals. VITAL-COMS supports these objectives by promoting in-depth patient discussions encompassing eating behaviors, appetite regulation, stress management, and sleep hygiene. Furthermore, the tool’s inherent adaptability allows for future customization, including scenario diversification and integration into broader health care curricula.

Although most participants completed the VITAL-COMS tool within 20‐30 minutes, one participant spent over 40 minutes using it. This longer duration likely reflects individual variation in communication style and clinical practice. In this case, the participant was an experienced dietitian working in secondary care, where consultations may be longer and more complex. Compared to others, the participant incorporated more patient-centered techniques, including taking a detailed weight history and using more open-ended questions to explore the patient’s perspective. This suggests that while the tool is generally time-efficient, practitioners with more in-depth communication approaches or working in specialist settings may require additional time. It highlights the flexibility of VITAL-COMS to adapt to different clinical styles and patient needs, rather than being a rigid script.

The observed minority of participants who expressed negative feedback regarding the training modality mirrors findings from other studies evaluating digital simulation and virtual reality-based interventions, where user preferences and technological comfort levels exhibit heterogeneity. As demonstrated by Chang et al (2023) [50], perceived ease of use and digital confidence significantly influence the acceptance and usage of simulation-based training tools. This observation is consistent with the technology acceptance model [40], which posits that perceived usefulness and usability are critical determinants of technology adoption. Consequently, the implementation of hybrid learning models that integrate digital and interpersonal learning strategies may enhance engagement and satisfaction across diverse learner profiles.

### Comparison With Previous Work

VITAL-COMS represents a novel, skills-based, communication-focused digital simulation tool specifically designed, tested, and evaluated for weight-related conversations. This innovation builds upon the growing body of research demonstrating the efficacy and acceptability of virtual character or virtual human interventions in health care communication training [51-53]. The scalable, immersive, and interactive nature of VITAL-COMS aligns with recent advancements in this area. Ryan et al (2024) [53] established a behavioral framework delineating essential training needs for health care professionals in weight-related communication and obesity science, many of which were also evident in the design of VITAL-COMS. However, VITAL-COMS distinguishes itself through its robust training needs analysis [30,31] and nuanced approach to modality (instructional videos, repetition, and guided feedback) and evaluation. This comprehensive methodology facilitates a more targeted and effective learning experience compared to recent progress.

Significant gaps remain in scalable weight-related communication skills training, particularly given the urgent need to address the rising prevalence of obesity [54,55]. This is shown through the current most available digital learning approaches as they often exhibit limitations in their scope and delivery. For instance, Logue et al [56], ‘Small talk, big difference’ intervention, while grounded in the COM-B behavioral theory, provided only a single one-hour online session and lacked opportunities for practical skill development and reflective learning. Similarly, the INTERACT study in Germany [57], which incorporated eLearning and motivational interviewing content, failed to demonstrate sustained improvements in key patient outcomes. In contrast, VITAL-COMS emphasizes experiential learning through practice, repetition, and reflection. Developed using the Unity 3D simulation platform, VITAL-COMS facilitates ongoing development of nuanced communication strategies and allows for adaptability to a wider range of patient scenarios. This focus on experiential learning represents a significant advancement in equipping health care professionals to address weight stigma and engage in sensitive weight discussions with confidence. Comparative platforms such as the Royal College of GPs’ obesity hub [58] and the global SCOPE training program [59] offer valuable educational material. However, these platforms typically lack immersive and interactive components. The inclusion of hands-on, realistic role-play with VITAL-COMS distinguishes it from these existing resources, thereby enhancing its potential for impactful translation into clinical practice.

### Strengths and Limitations

The limited sample size, combined with recruitment challenges among students, likely exacerbated by the COVID-19 pandemic and the sensitive nature of the training topic, constrains the generalizability of the findings. Furthermore, while the communication self-assessment tool demonstrated preliminary promise, it requires further validation against established competency frameworks. The reliance on self-report measures, while common in communication skills training, introduces recognized limitations associated with subjective reporting bias. To mitigate potential inconsistencies arising from human facilitation, a recognized limitation of WoZ methodologies, the research team implemented a rigorous protocol to standardize instructions and ensure a consistent participant experience. This approach adhered to core principles of WoZ design experiments. Nevertheless, the potential for residual variability in human facilitation remains a consideration when interpreting the study’s findings.

### Future Work

VITAL-COMS, as an advanced prototype, used a WoZ methodology to simulate real-time natural language interaction with virtual patient characters, a functionality that was not readily available at the time of development. The findings of this feasibility study provide valuable insights for the potential integration of emerging technologies, such as generative AI chatbots, which now offer real-time natural language interaction and feedback capabilities. These advancements present a promising alternative to the WoZ approach, potentially enabling broader and more scalable training delivery. Furthermore, the feasibility of automated AI-driven assessment should be explored in future iterations of VITAL-COMS, addressing the inherent limitations associated with self-report assessment measures. Future development of VITAL-COMS includes expanding the range of characters and scenarios, adapting content for different health care settings and professional groups, incorporating advanced technologies, and ultimately, informing the development of the next iteration of the VITAL-COMS prototype driven by generative AI.

### Conclusion

This study found that VITAL-COMS was acceptable to a diverse group of HCPs and it improved weight-related communication skills. While most participants were enthusiastic about the real-time simulation, some also recognized its potential value when used alongside more traditional learning methods. Further development is needed to tailor the training to different professional groups. The findings from this study will be used to inform the next iteration of the VITALS-COMS communication skills training tool driven by generative AI.

## Supplementary material

10.2196/65949 Multimedia Appendix 1 Multimedia demonstration of VITAL-COMS (virtual training and assessment for communication skills), how the health care professional talks to the virtual character.

10.2196/65949 Multimedia Appendix 2 Presentation from the International Conference on Communication in Healthcare (2022), explaining how VITAL-COMS works and how Wizard of Oz is used as part of the design.

10.2196/65949 Multimedia Appendix 3 Self-reported knowledge assessment questionnaire on weight-related communication skills.

10.2196/65949 Multimedia Appendix 4 Example educational video 1 used in VITAL-COMS (virtual training and assessment for communication skills) to educate on obesity science and communication.

10.2196/65949 Multimedia Appendix 5 Example educational video 2 used in VITAL-COMS (virtual training and assessment for communication skills) to educate on weight stigma.

## Abbreviations

AI: artificial intelligence
COM-B: capability, opportunity, and motivation for behavior change
CONSORT: Consolidated Standards of Reporting Trials
HCP: health care professional
VITAL-COMS: virtual training and assessment for communication skills
WoZ: Wizard of Oz
