# Supplementary material for: A Virtual Simulator to Improve Weight-Related Communication Skills for Health Care Professionals: Mixed Methods Pre-Post Pilot Feasibility Study
Source: JMIR Med Educ. 2025 Aug 15;11:e65949. doi: 10.2196/65949 (PMC12356524; doi:10.2196/65949)
Supplement: Multimedia Appendix 2 [file mededu-v11-e65949-s002.pptx]

## Slide 1
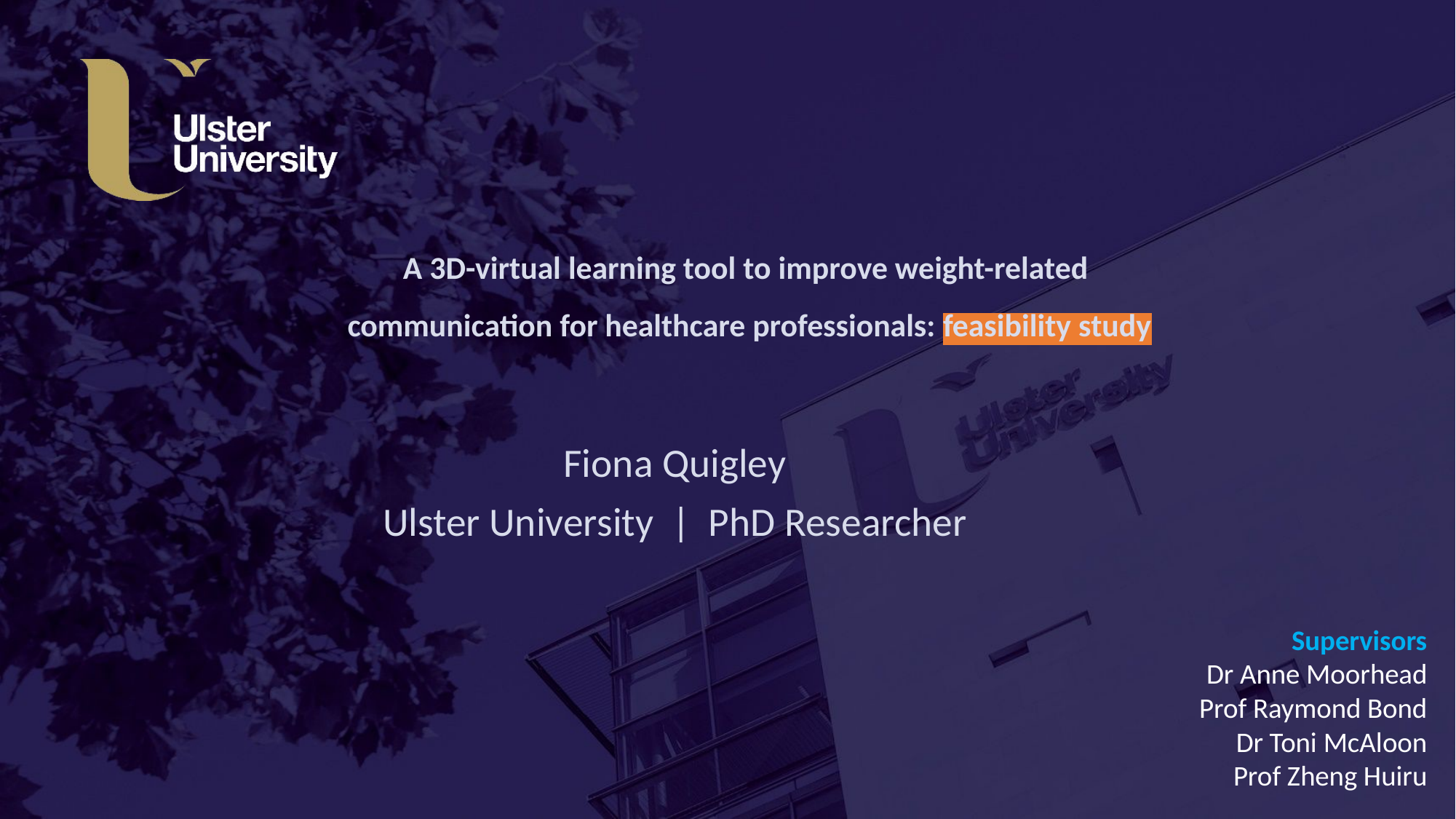

# A 3D-virtual learning tool to improve weight-related communication for healthcare professionals: feasibility study
Fiona Quigley
Ulster University | PhD Researcher
Supervisors
Dr Anne Moorhead
Prof Raymond Bond
Dr Toni McAloon
Prof Zheng Huiru

## Slide 2
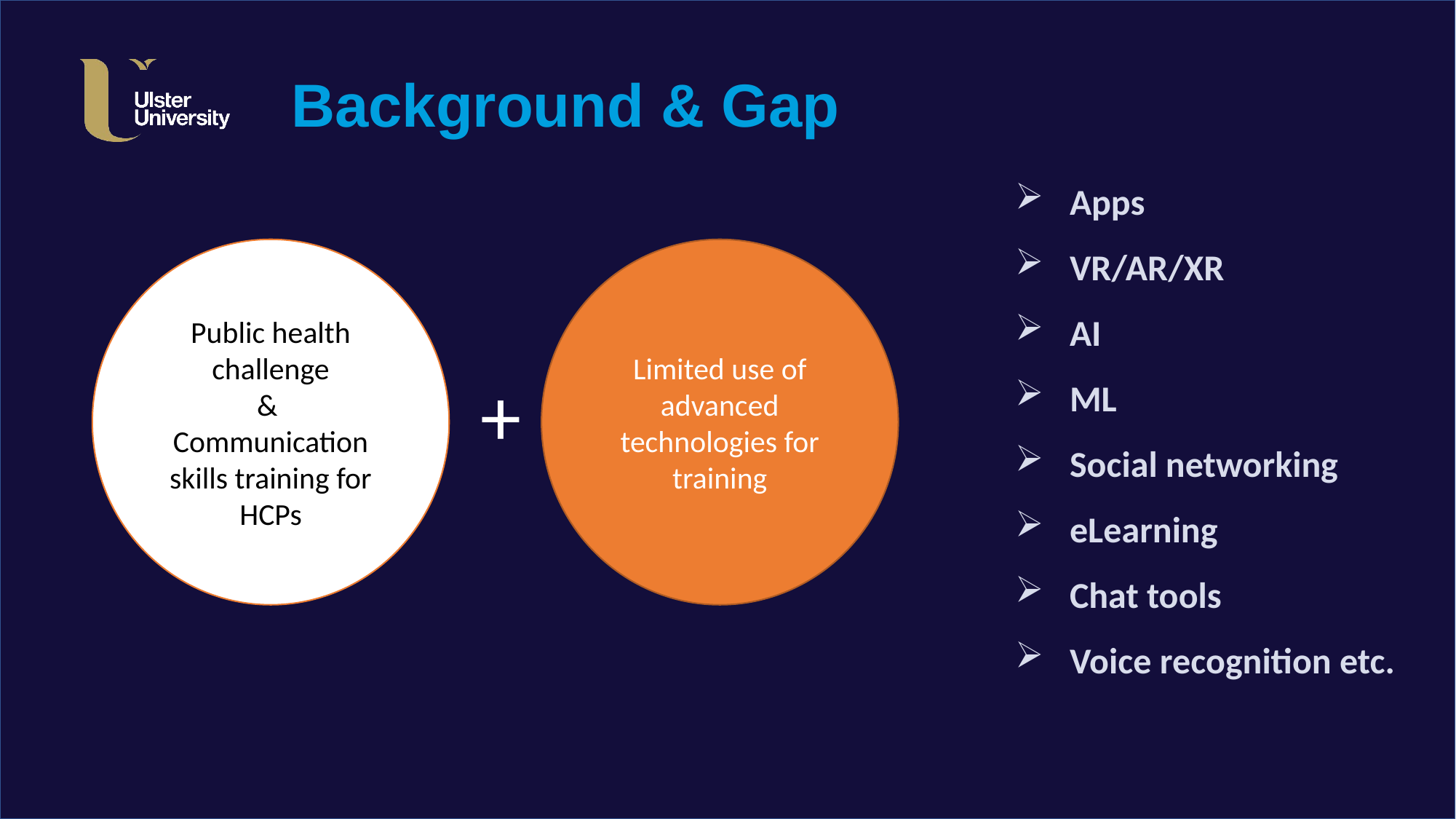

# Background & Gap
Apps
VR/AR/XR
AI
ML
Social networking
eLearning
Chat tools
Voice recognition etc.
Limited use of advanced technologies for training
Public health challenge
&
Communication skills training for HCPs
+

## Slide 3
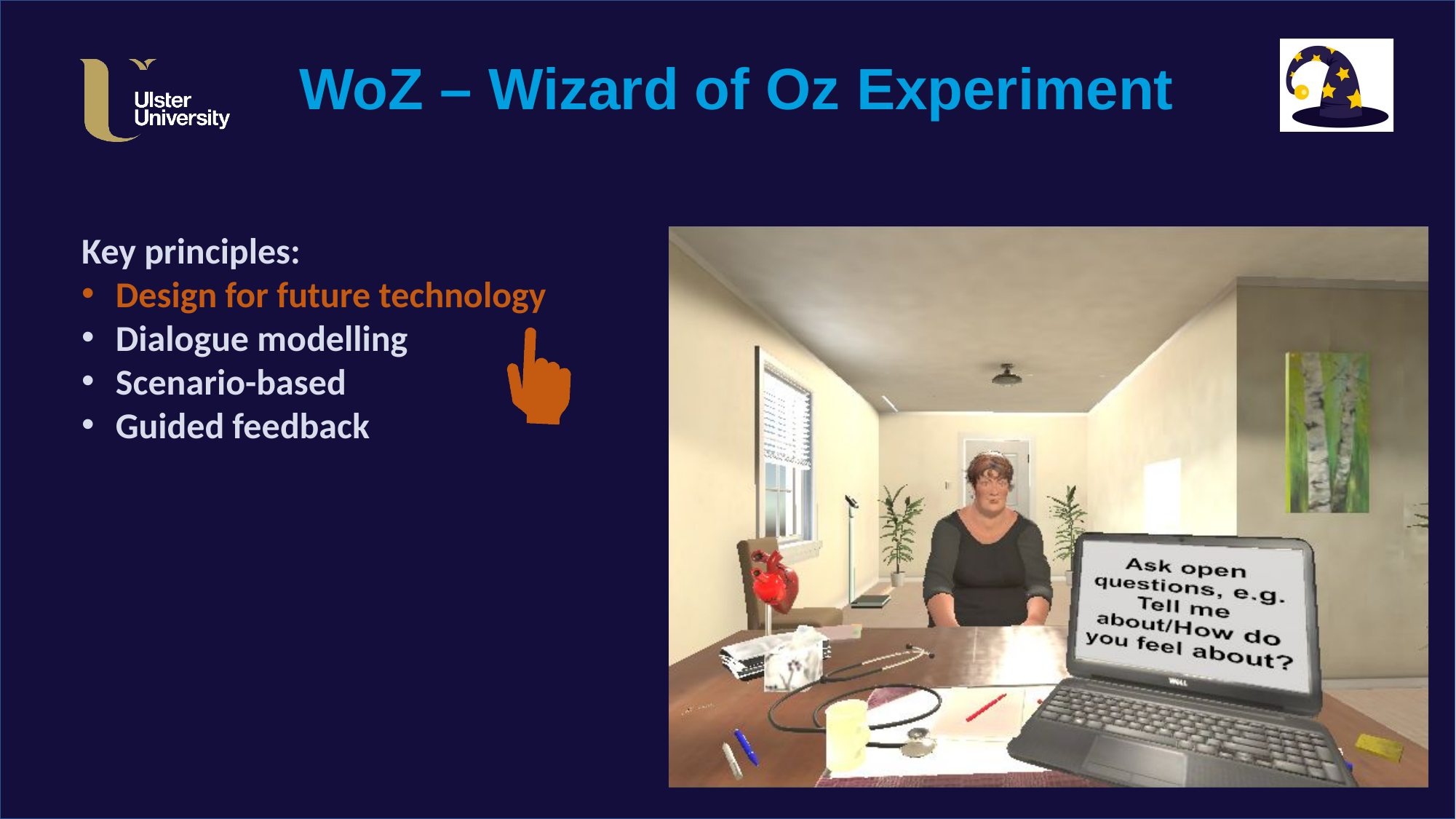

WoZ – Wizard of Oz Experiment
Key principles:
Design for future technology
Dialogue modelling
Scenario-based
Guided feedback

## Slide 4
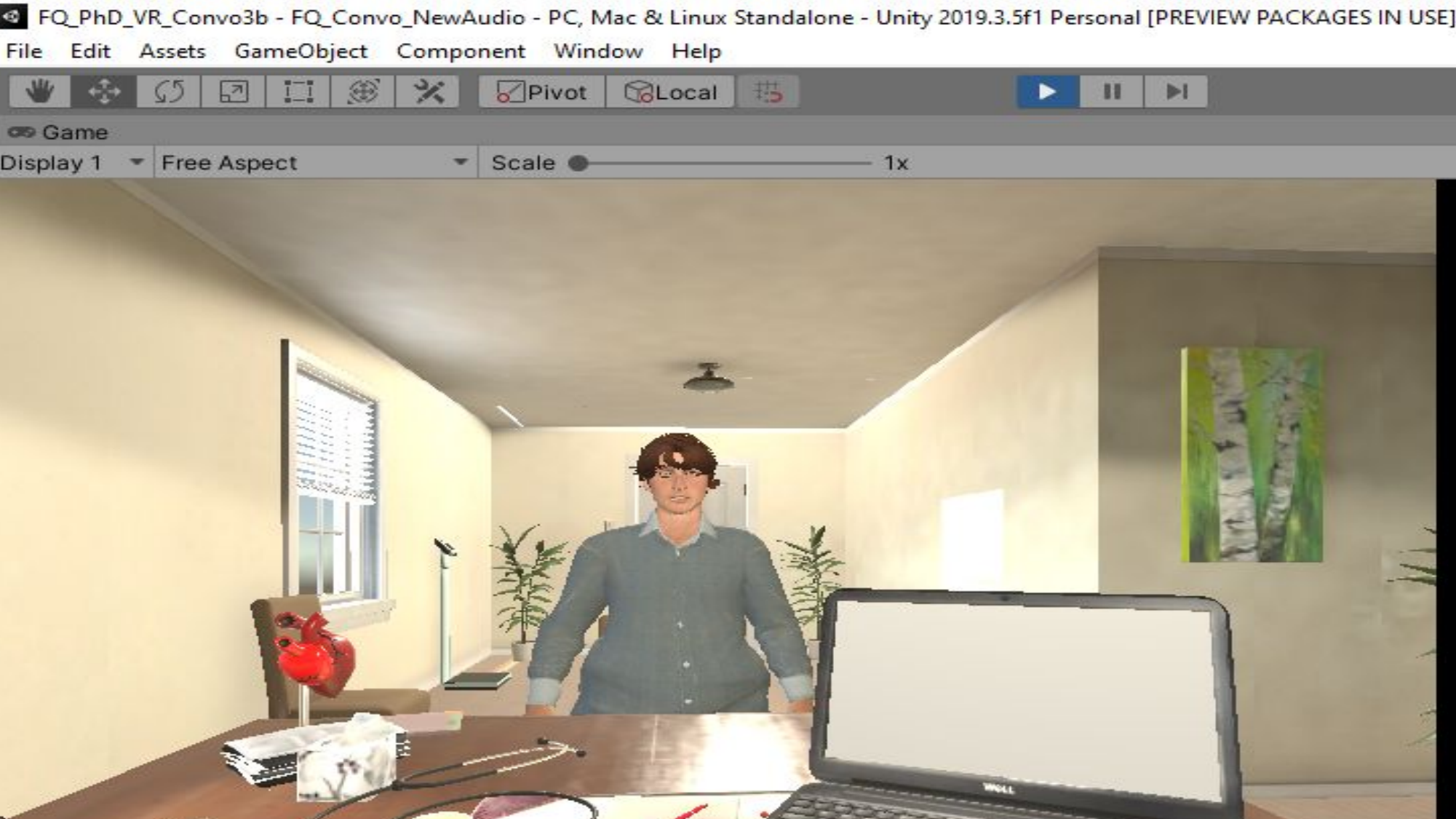

# Training Scenarios 1/2
Bob
Scenario 2: Gaining weight
(Age 35, BMI 32 ( 41)

## Slide 5
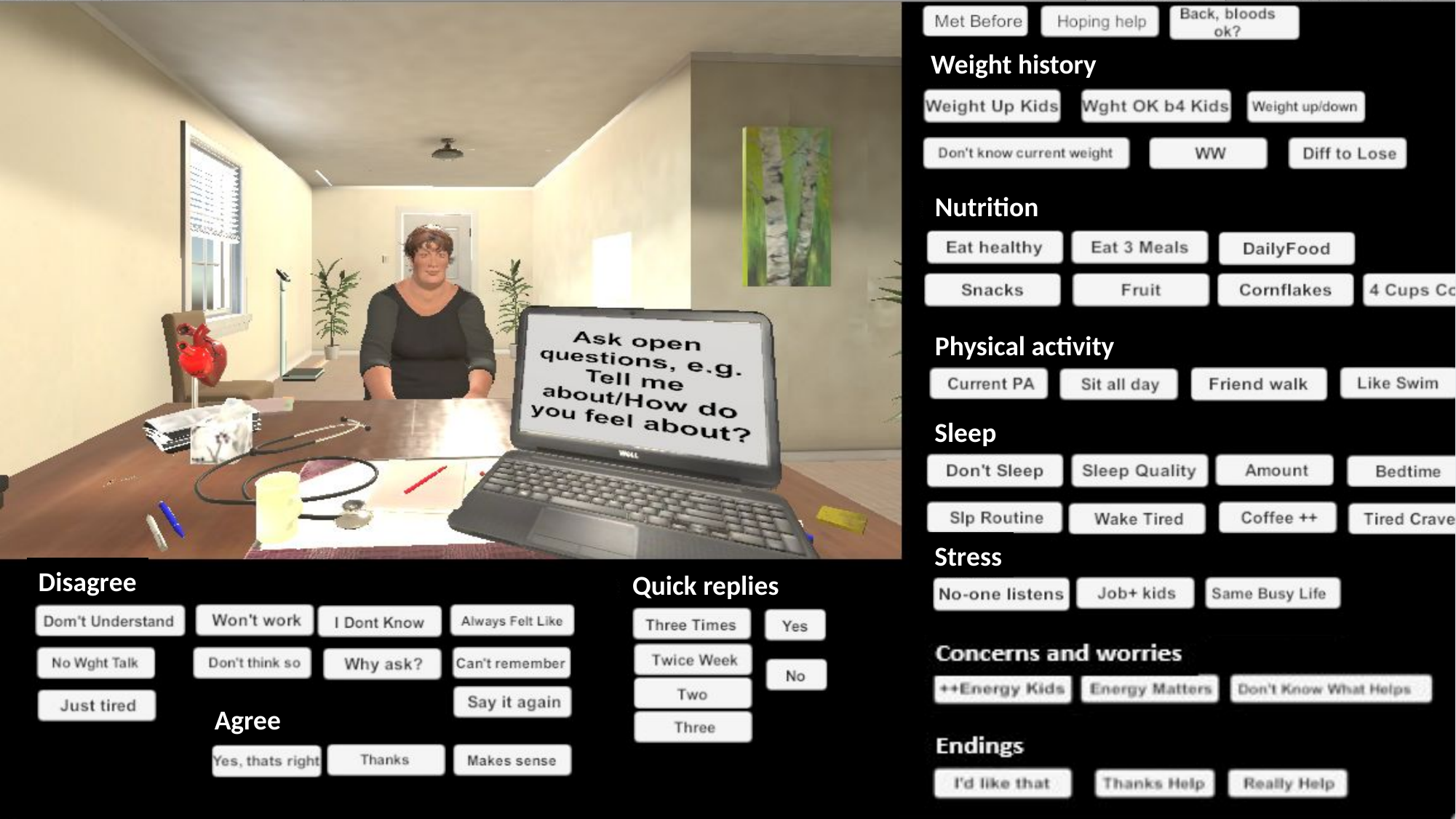

Weight history
Nutrition
Physical activity
Sleep
Stress
Disagree
Quick replies
Agree
WoZ – Wizard of Oz Experiment
Concerns and worries
Endings

## Slide 6
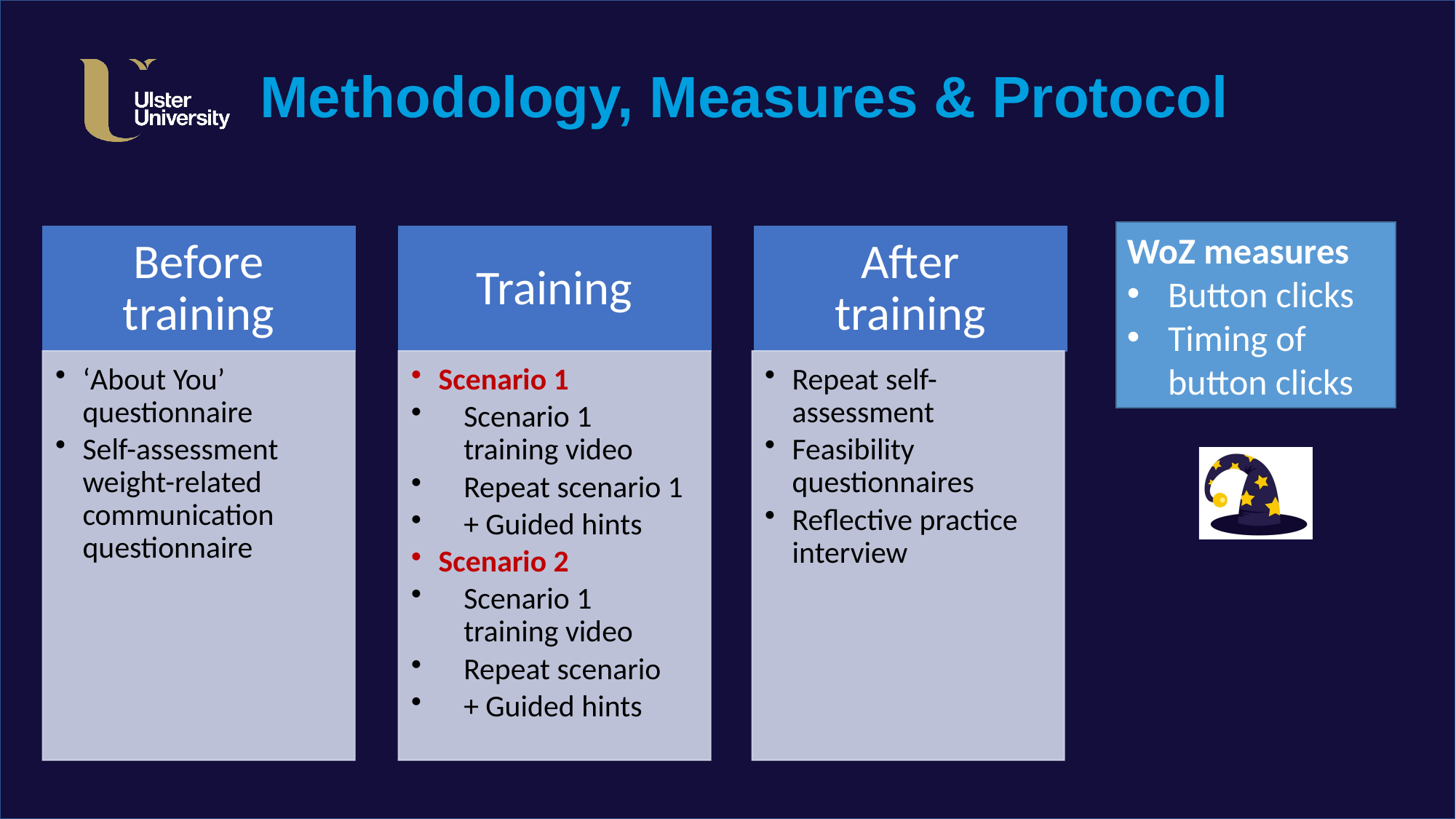

# Methodology, Measures & Protocol
WoZ measures
Button clicks
Timing of button clicks

## Slide 7
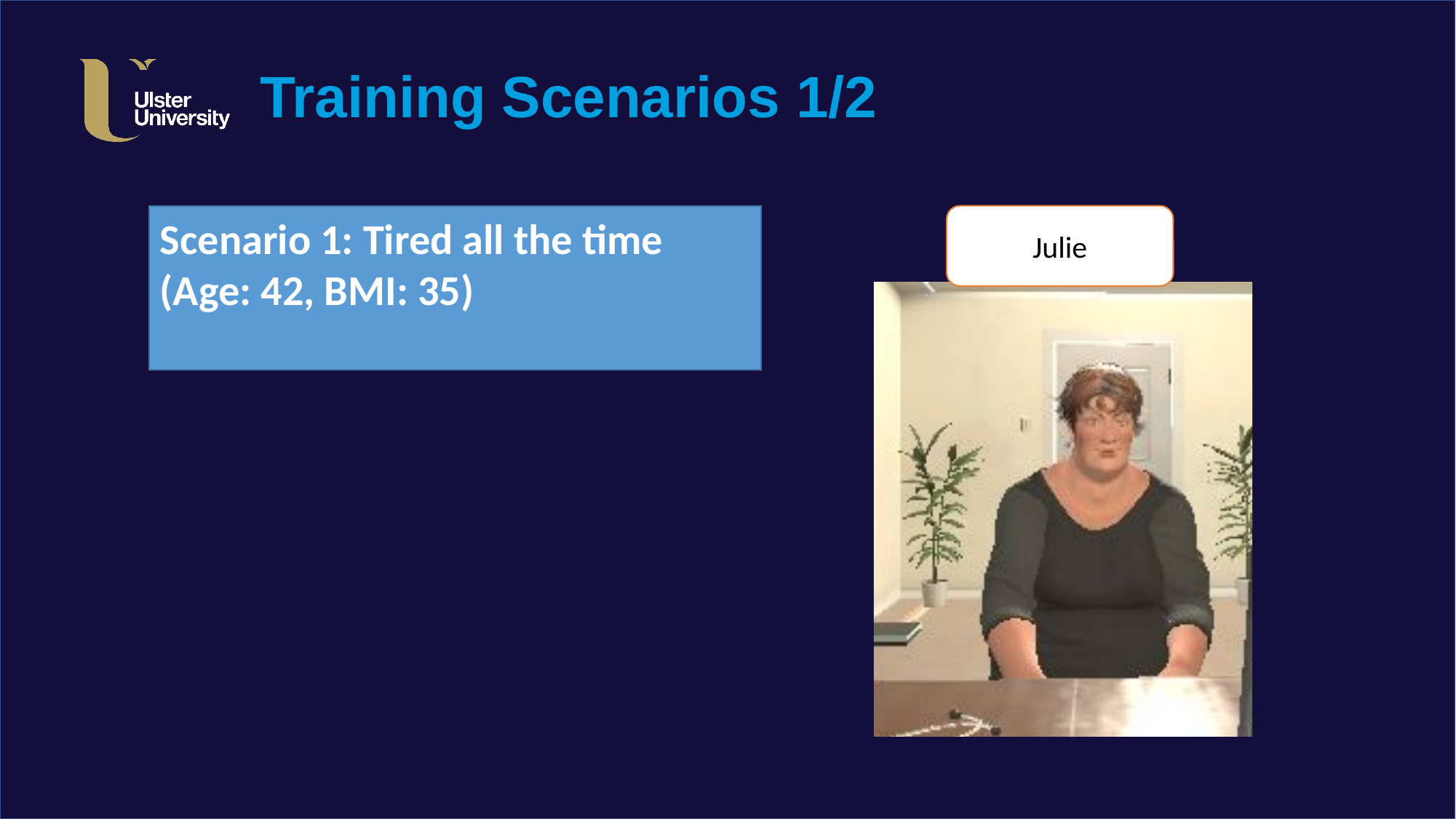

# Training Scenarios 1/2
Julie
Scenario 1: Tired all the time (Age: 42, BMI: 35)

## Slide 8
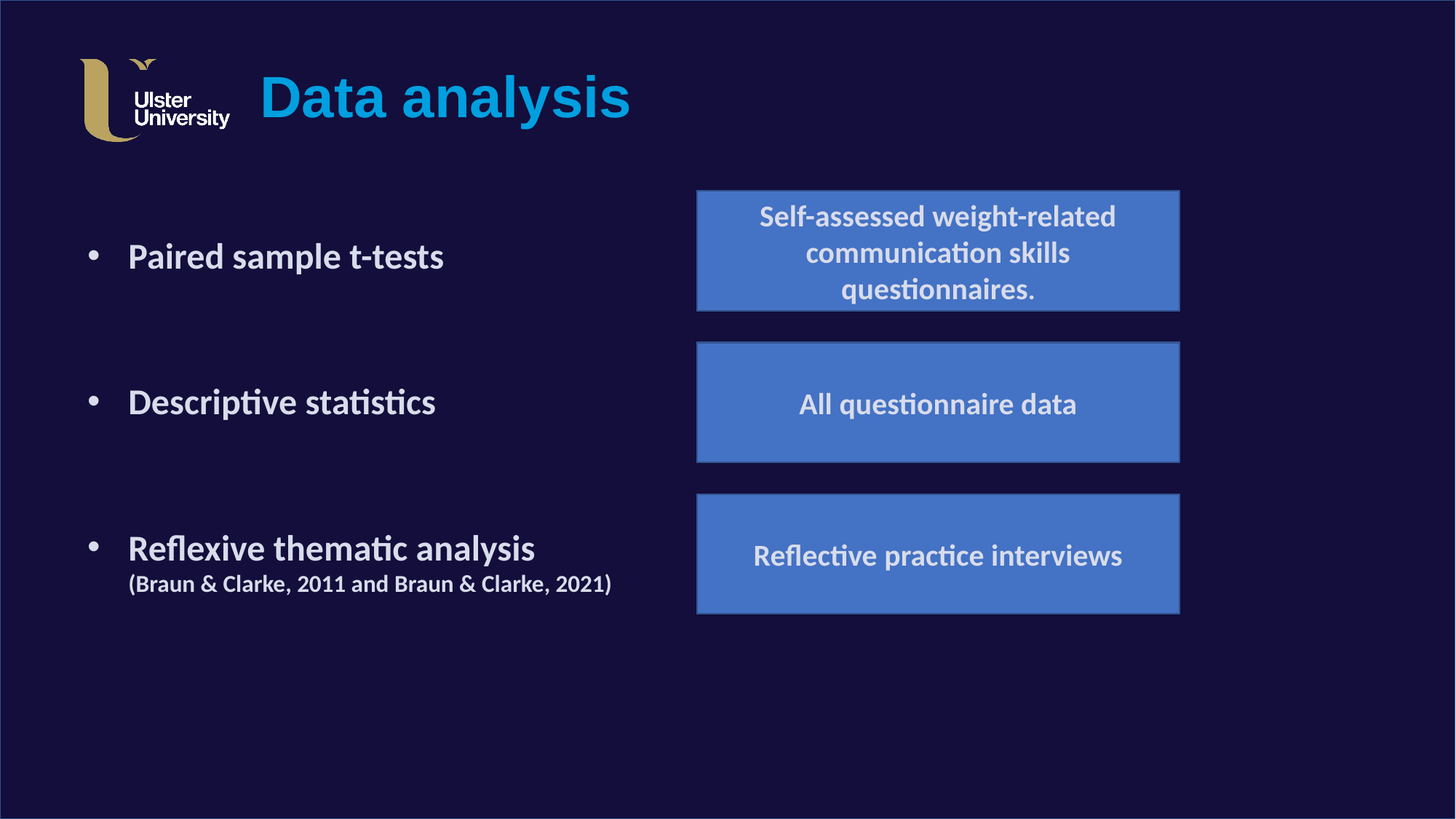

# Data analysis
Self-assessed weight-related communication skills questionnaires.
Paired sample t-tests
Descriptive statistics
Reflexive thematic analysis
(Braun & Clarke, 2011 and Braun & Clarke, 2021)
All questionnaire data
Reflective practice interviews

## Slide 9
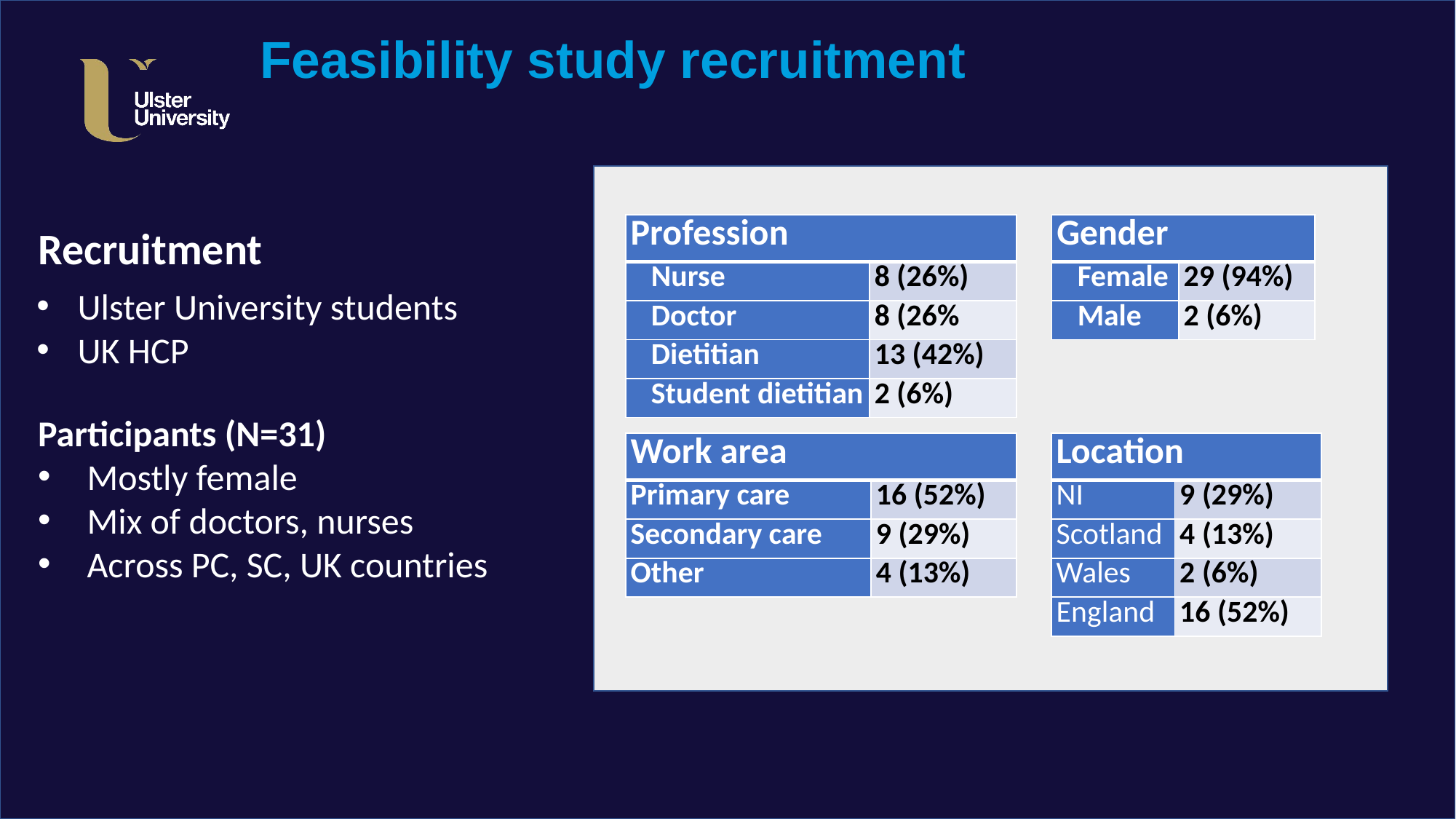

# Feasibility study recruitment
| Profession | |
| --- | --- |
| Nurse | 8 (26%) |
| Doctor | 8 (26% |
| Dietitian | 13 (42%) |
| Student dietitian | 2 (6%) |
| Gender | |
| --- | --- |
| Female | 29 (94%) |
| Male | 2 (6%) |
Recruitment
Ulster University students
UK HCP
Participants (N=31)
Mostly female
Mix of doctors, nurses
Across PC, SC, UK countries
| Work area | |
| --- | --- |
| Primary care | 16 (52%) |
| Secondary care | 9 (29%) |
| Other | 4 (13%) |
| Location | |
| --- | --- |
| NI | 9 (29%) |
| Scotland | 4 (13%) |
| Wales | 2 (6%) |
| England | 16 (52%) |

## Slide 10
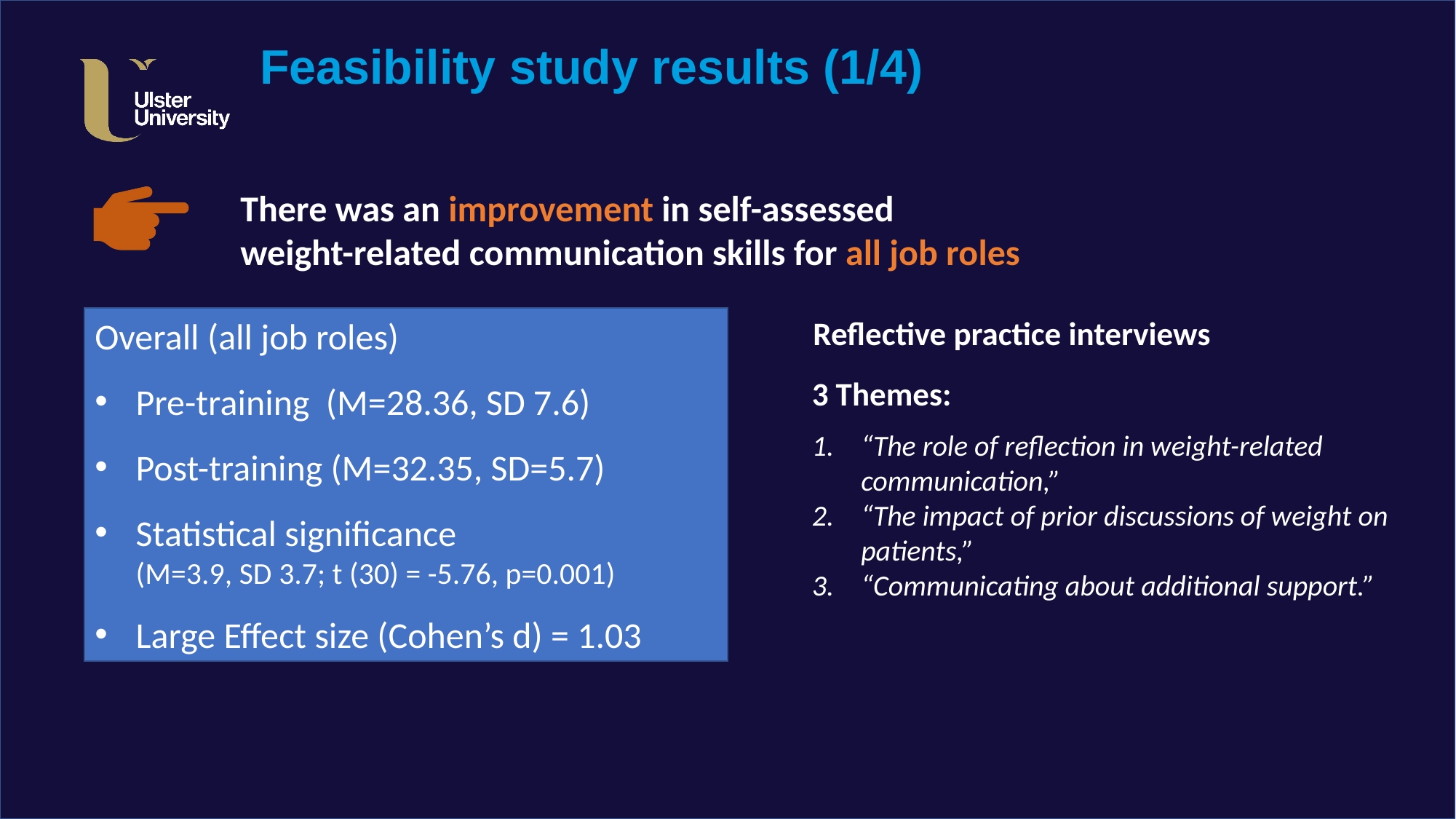

# Feasibility study results (1/4)
There was an improvement in self-assessed
weight-related communication skills for all job roles
Reflective practice interviews
Overall (all job roles)
Pre-training (M=28.36, SD 7.6)
Post-training (M=32.35, SD=5.7)
Statistical significance
(M=3.9, SD 3.7; t (30) = -5.76, p=0.001)
Large Effect size (Cohen’s d) = 1.03
3 Themes:
“The role of reflection in weight-related communication,”
“The impact of prior discussions of weight on patients,”
“Communicating about additional support.”

## Slide 11
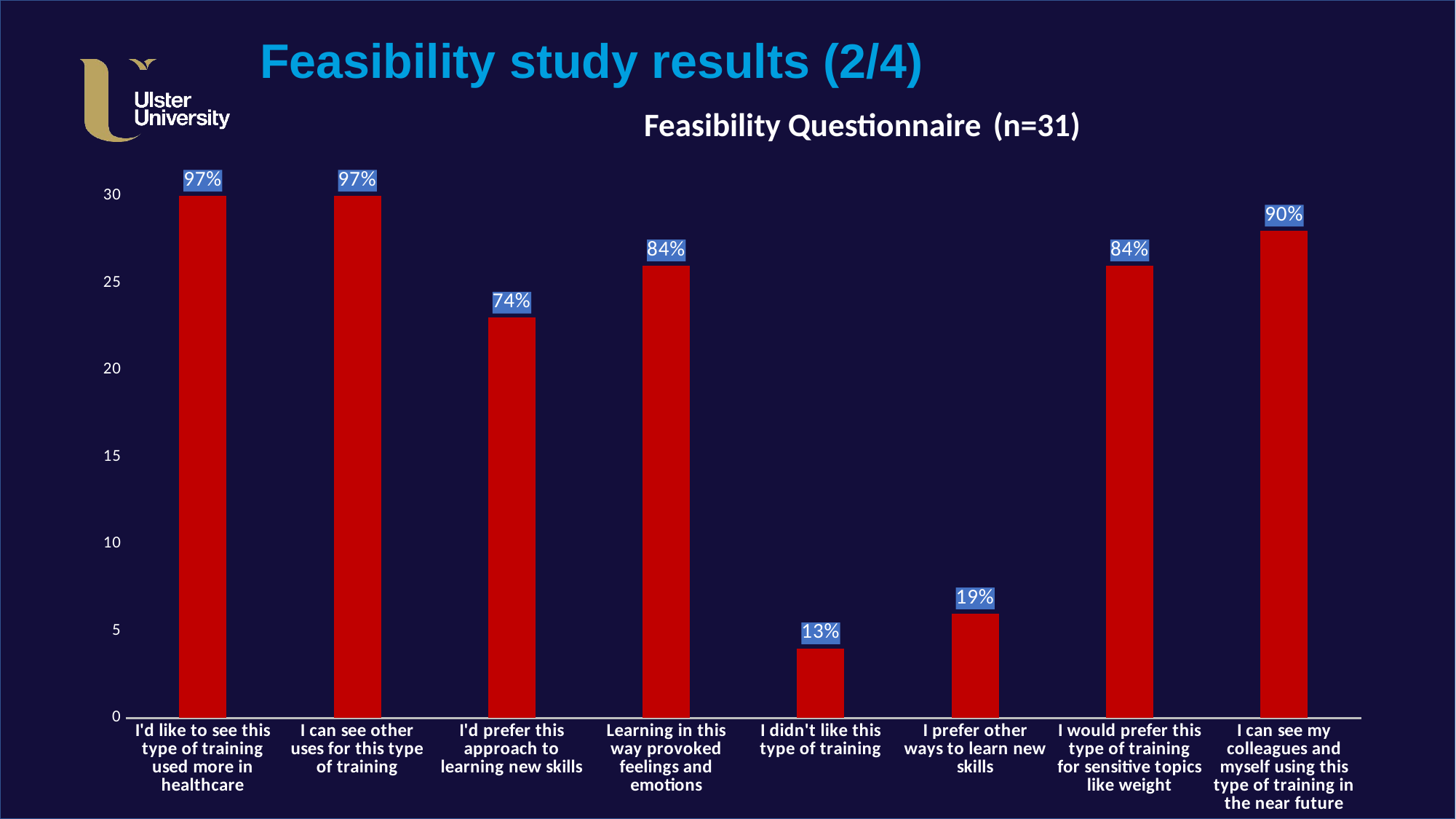

Feasibility study results (2/4)
Feasibility Questionnaire
(n=31)
### Chart
| Category | Yes |
|---|---|
| I'd like to see this type of training used more in healthcare | 30.0 |
| I can see other uses for this type of training | 30.0 |
| I'd prefer this approach to learning new skills | 23.0 |
| Learning in this way provoked feelings and emotions | 26.0 |
| I didn't like this type of training | 4.0 |
| I prefer other ways to learn new skills | 6.0 |
| I would prefer this type of training for sensitive topics like weight | 26.0 |
| I can see my colleagues and myself using this type of training in the near future | 28.0 |

## Slide 12
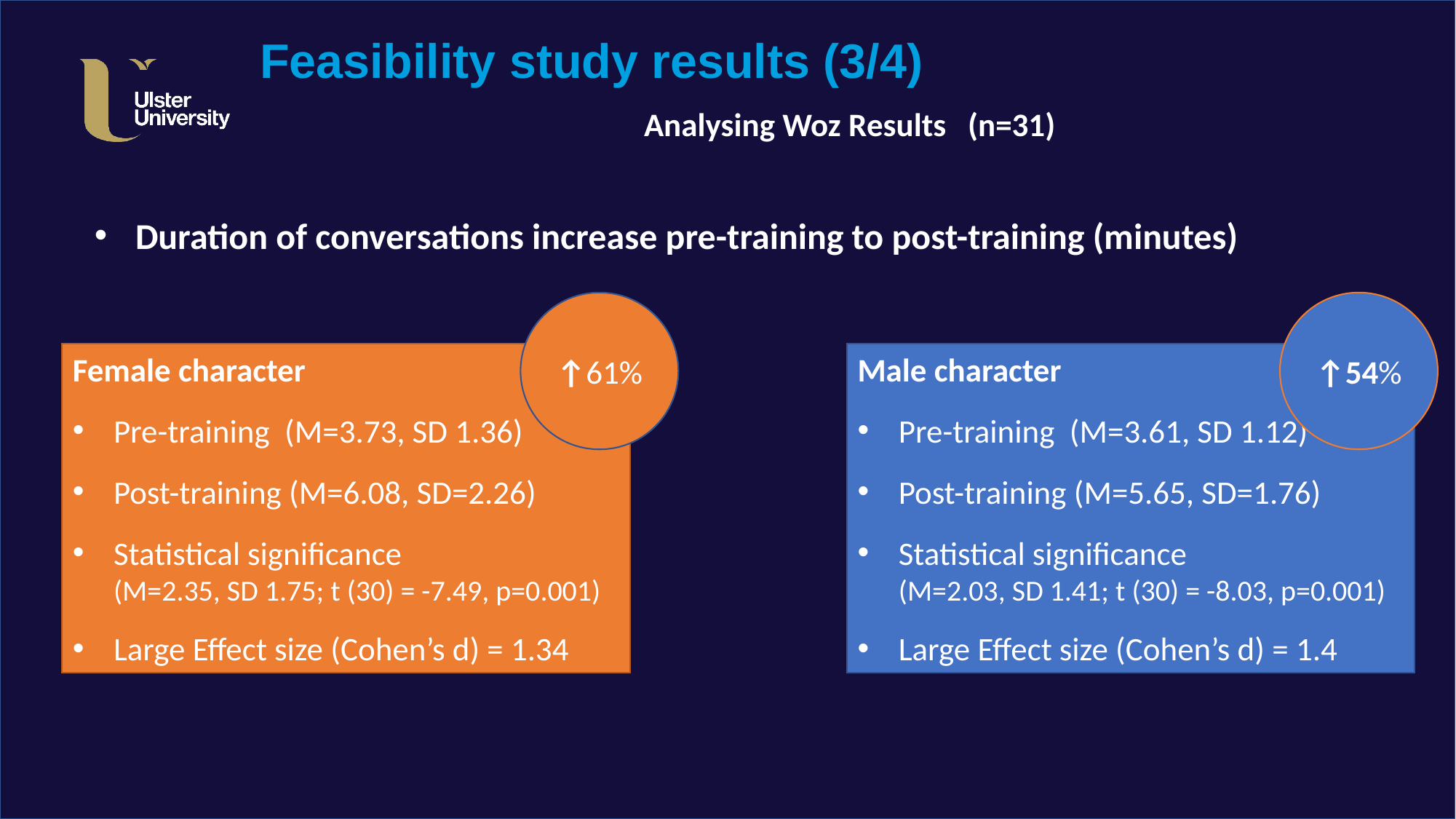

Feasibility study results (3/4)
Analysing Woz Results
(n=31)
Duration of conversations increase pre-training to post-training (minutes)
↑61%
↑54%
Female character
Pre-training (M=3.73, SD 1.36)
Post-training (M=6.08, SD=2.26)
Statistical significance
(M=2.35, SD 1.75; t (30) = -7.49, p=0.001)
Large Effect size (Cohen’s d) = 1.34
Male character
Pre-training (M=3.61, SD 1.12)
Post-training (M=5.65, SD=1.76)
Statistical significance
(M=2.03, SD 1.41; t (30) = -8.03, p=0.001)
Large Effect size (Cohen’s d) = 1.4

## Slide 13
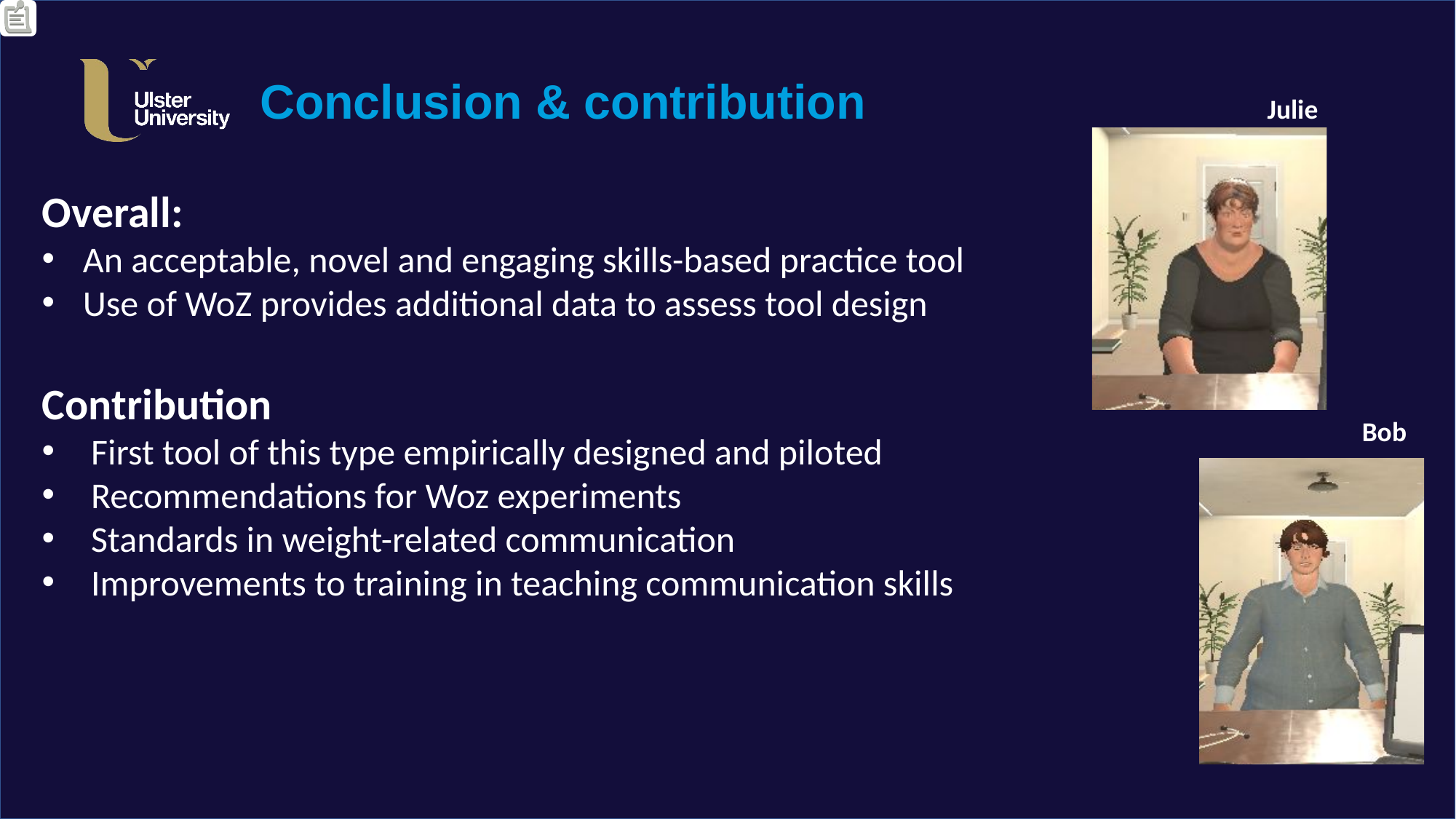

# Conclusion & contribution
Julie
Overall:
An acceptable, novel and engaging skills-based practice tool
Use of WoZ provides additional data to assess tool design
Contribution
First tool of this type empirically designed and piloted
Recommendations for Woz experiments
Standards in weight-related communication
Improvements to training in teaching communication skills
Bob
