# Supplementary figures and images for: A Virtual Simulator to Improve Weight-Related Communication Skills for Health Care Professionals: Mixed Methods Pre-Post Pilot Feasibility Study
Source: JMIR Med Educ. 2025 Aug 15;11:e65949. doi: 10.2196/65949 (PMC12356524; doi:10.2196/65949)

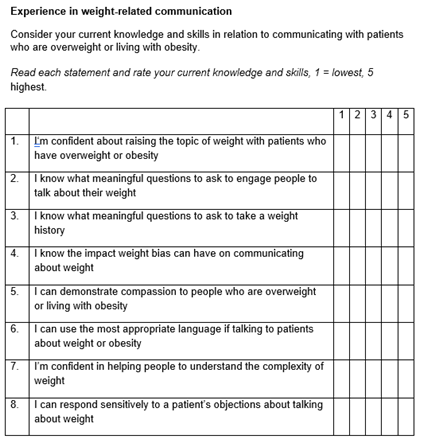

Supplement: Multimedia Appendix 3 [file mededu-v11-e65949-s003.png]
